# Stream Hydrological Fragmentation Drives Bacterioplankton Community Composition

**DOI:** 10.1371/journal.pone.0064109

**Published:** 2013-05-31

**Authors:** Stefano Fazi, Eusebi Vázquez, Emilio O. Casamayor, Stefano Amalfitano, Andrea Butturini

**Affiliations:** 1 Water Research Institute, National Research Council of Italy (IRSA - CNR), Monterotondo, Roma, Italy; 2 Department d'Ecologia, Facultat de Biologia, Universitat de Barcelona (UB), Barcelona, Spain; 3 Department of Continental Ecology-Biodiversity and Biogeodynamics Interactions, Centro de Estudios Avanzados de Blanes, Consejo Superior de Investigaciones Científicas (CEAB-CSIC), Blanes, Girona, Spain; Wageningen University, The Netherlands

## Abstract

In Mediterranean intermittent streams, the hydrological fragmentation in summer and the successive water flow re-convergence in autumn allow exploring how local processes shape the microbial community within the same habitat. The objectives of this study were to determine how bacterial community composition responded to hydrological fragmentation in summer, and to evaluate whether the seasonal shifts in community composition predominate over the effects of episodic habitat fragmentation. The bacterial community was assessed along the intermittent stream Fuirosos (Spain), at different levels of phylogenetic resolution by in situ hybridization, fingerprinting, and 16S rRNA gene sequencing. The hydrological fragmentation of the stream network strongly altered the biogeochemical conditions with the depletion of oxidized solutes and caused changes in dissolved organic carbon characteristics. In the isolated ponds, beta-*Proteobacteria* and *Actinobacteria* increased their abundance with a gradual reduction of the alpha-diversity as pond isolation time increased. Moreover, fingerprinting analysis clearly showed a shift in community composition between summer and autumn. In the context of a seasonal shift, the temporary stream fragmentation simultaneously reduced the microbial dispersion and affected local environmental conditions (shift in redox regime and quality of the dissolved organic matter) tightly shaping the bacterioplankton community composition.

## Introduction

Microbial communities can exhibit spatial variability at scales ranging from millimetres to thousands of kilometres [1]. Understanding the mechanisms governing such spatial distribution is a key issue for elucidating the extent, specificity and stability of microbial associations and the implications for ecosystem functioning [2]–[5]. Microbial ecologists have recently started to examine the role of dispersal in shaping community similarities on large spatial scales [6]–[8]. Moreover, the microbial responses to local environmental stresses have to be carefully considered to properly interpret the dynamics of the microbial world [9].

The temporal and spatial variability found in bacterial community composition is mainly driven by species sorting and fast local growth, which counterbalance cell dispersion [10]–[12]. Both local interactions (e.g. within/between species, between species and the environment) and regional processes (e.g. dispersal) influence local community assembly [4]. High dispersal rates can lead to a continuous, worldwide supply of taxa that can be found even in less suitable habitats [13]. Local environmental characteristics are relatively more important when variation between sites increases [7], [14], [15,]. To gain a better understanding on how the local environment shapes bacterial communities it is necessary to address the proper spatial resolution at which microorganisms assemble into local communities, thus minimising regional effects at larger spatial scale [6], [16].

In the case of freshwater environments, most studies tended to focus on either similar habitats across different spatial scales or interconnected habitats, reaching different conclusions on the importance of environmental factors [11], [17] and geographic distance [8], [18]–[20]. So far, few studies have focused on assessing microbial assemblages when a habitat undergoes a gradual fragmentation, which interrupts the flow of carbon and energy, the dispersion of biota, and causes a marked environmental heterogeneity [21], [22]. In intermittent streams, hydrological fragmentation in summer and water flow re-convergence in autumn could provide the opportunity to explore how local processes shape a microbial community within the same habitat. Episodes of low flow fragment the hydrological stream network into a patched landscape of unconnected standing water bodies. As a consequence, fragmentation increases the environmental heterogeneity and decreases hydrological connectivity and the potential for bacterial cell dispersal.

We designed a field study following a gradient approach (from flowing to stagnant waters, in ponds disconnected at different times) that provides a framework for discussing how bacterial community structure relates to water biogeochemistry during the stream habitat fragmentation episodes. To avoid confounding regional influences [14], we selected a small semi-pristine intermittent Mediterranean stream (Fuirosos, Spain) and microbial assemblages were assessed along the whole stream network. During summer, the streambed was completely dry except in the five locations where water samples were collected. A second sampling campaign was carried out in the same sites when the stream connectivity was re-established in autumn. The hydrology and the water chemistry were characterised and the bacterial assemblages were assessed at different levels of phylogenetic resolution by Fluorescence in situ Hybridization Catalysed Reported Deposition (CARD-FISH) and by Denaturing Gradient Gel Electrophoresis (DGGE) and 16S rRNA gene sequencing.

In this study, we aimed to determine (i) how the abundance and composition of bacterioplankton communities respond to the hydrological and biogeochemical changes when the stream shifts between free-flowing and fragmented non-flowing conditions in summer; and (ii) how the seasonal shift, in the transition from summer to autumn, affects the occurrence of the major bacterial species and predominates over the effects of episodic habitat fragmentation.

## Results

### Hydrological and chemical dynamics

**Figure 1:**
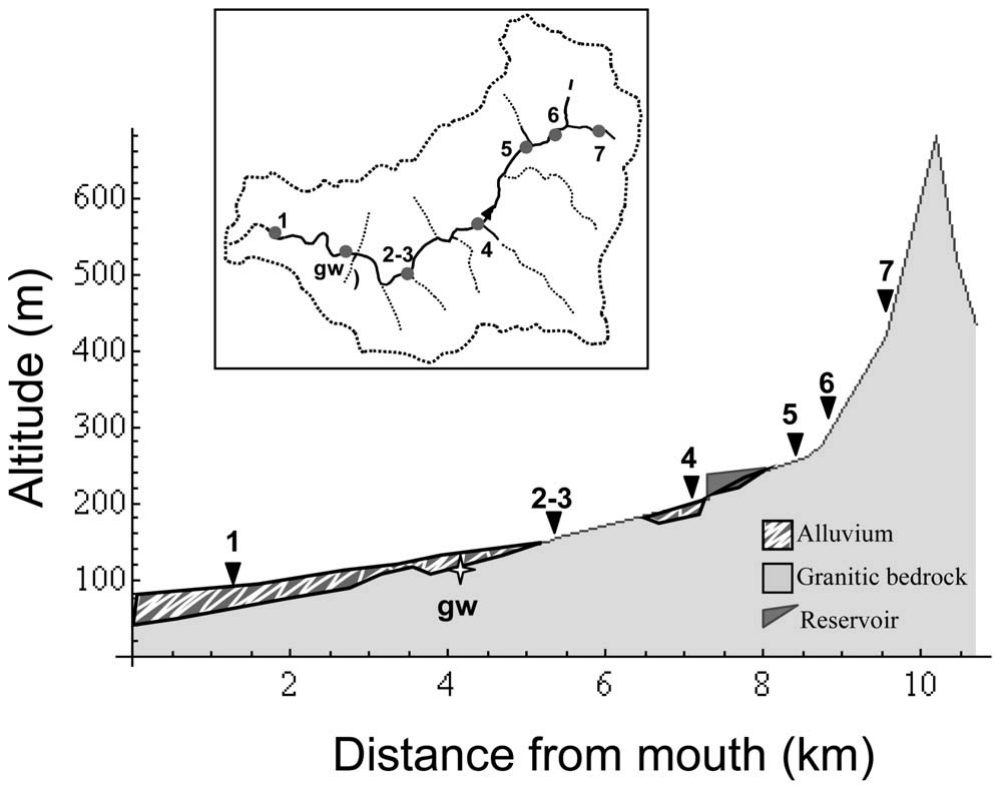
Sampling point locations. Sampling point locations along the longitudinal-altitudinal profile and the fluvial network (inset). Triangles: surface waters. Star: riparian groundwater.

**Figure 2:**
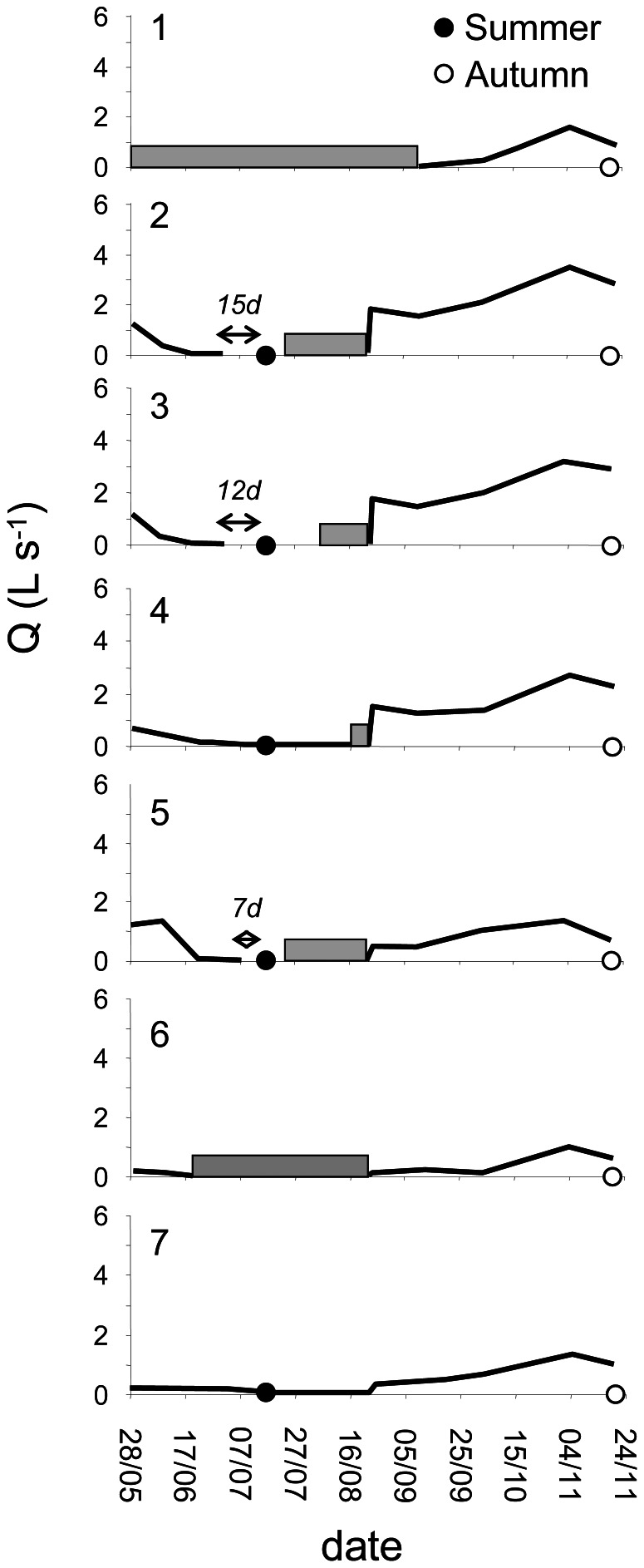
Water discharge in the sampling points. Discharge from May to November in the sampling points (1–7). (•) = summer sampling; (○) = autumn sampling; (↔) = period between water segregation in the pond (no flow) and sampling time; gray bars indicate the period of complete dryness of the stream reach.

Hydrological fragmentation enhanced anaerobic conditions due to both depletion of oxidised solutes (O_2_, N-NO_3_, SO_4_) and accumulation of reduced solutes such as dissolved organic carbon (DOC), dissolved organic nitrogen (DON) and N-NH_4_ in isolated ponds (i.e. sites 2, 3 and 5) (Fig. 1 and 2; Table 1). The lowest values of the Chemical Index (CI<0.3), used to describe the degree of the aerobic/anaerobic conditions in the waters bodies, were found in the isolated ponds, while the highest values (CI>3) were observed in flowing waters (both in summer and in autumn) and in groundwater. An inverse relationship between the CI and the Pond Isolation Time (PIT) was observed (r = 0.92, p<0.05, df = 3).

**Table 1:** Water physical and chemical characteristics in sampling points.

		Summer								Autumn						
		1	2	3	4	5	6	7	gw	1	2–3	4	5	6	7	gw
**Water physical and chemical characteristics**	PIT (d)	dry	15	12	0	7	dry	0	nm	nm	nm	nm	nm	nm	nm	nm
	Q (l/s)	dry	0	0	0.1	0	dry	0.2	nm	0.9	2.9	2.3	0.7	0.6	0.6	nm
	Temperature (°C)	dry	18.2	17.1	17.5	16.7	dry	18.0	17.0	11.3	11.3	11.4	9.5	11.4	9.6	14.8
	O_2_ (mg/l)	dry	0.5	1.0	1.5	2.0	dry	9.0	6.5	8.3	10.8	7.3	9.1	5.4	10.3	8.4
	pH	dry	7.3	7.3	6.9	7.3	dry	8.0	6.9	7.2	7.6	7.5	7.5	7.0	7.6	7.4
	EC (µS/cm)	dry	463	349	349	408	dry	161	387	265	267	220	258	274	164	419
	Chloride (mg/l)	dry	33.9	29.7	18.9	21.7	dry	14.1	20.2	26.3	23.5	20.5	21.6	23.2	14.2	23.2
			(*)	(*)	(*)	(*)		(*)	(*)	(*)	(*)	(*)	(*)	(*)	(*)	(*)
	SO_4_ (mg/l)	dry	4.7	3.6	2.8	10.3	dry	8.4	36.5	21.6	21.9	19.2	19.1	21.5	9.3	14.4
			(*)	(*)	(*)	(*)		(*)	(*)	(*)	(*)	(*)	(*)	(*)	(*)	(*)
	N-NO_3_ (mg/l)	dry	0.08	0.01	0.02	0.08	dry	0.23	1.31	0.25	0.01	0.12	0.34	0.74	0.04	0.02
			(*)	(0.001)	(0.004)	(*)		(0.060)	(*)	(0.050)	(0.010)	(*)	(*)	(*)	(0.030)	(0.060)
	N-NH_4_ (mg/l)	dry	13.00	1.89	0.10	1.55	dry	0.02	0.04	0.04	0.04	0.02	0.01	0.03	0.01	0.01
			(*)	(*)	(0.020)	(0.190)		(0.005)	(0.010)	(0.060)	(*)	(0.004)	(0.003)	(*)	(0.010)	(0.004)
	P-PO_4_ (mg/l)	dry	0.017	0.017	0.001	0.031	dry	0.017	0.008	0.001	0.001	0.001	0.001	0.001	0.001	0.001
			(*)	(*)	(*)	(*)		(*)	(*)	(*)	(*)	(*)	(*)	(*)	(*)	(*)
	CI	dry	−3.3	−0.6	2.7	0.3	dry	6.3	5.0	5.2	5.6	6.1	6.6	5.3	7.2	6.6
			(*)	(*)	(0.1)	(0.1)		(0.3)	(0.3)	(1.3)	(*)	(*)	(0.6)	(0.6)	(*)	(*)
**DOM characteristics**	DOC (mg/l)	dry	33.1	5.9	6.0	6.1	dry	1.8	1.7	1.7	2.8	4.1	2.0	2.2	1.9	1.1
			(*)	(*)	(*)	(*)		(*)	(0.60)	(0.02)	(*)	(*)	(*)	(*)	(0.41)	(*)
	DON (mg/l)	dry	1.31	0.14	0.29	0.87	dry	0.05	0.09	0.11	0.17	0.32	0.16	0.14	0.08	0.17
			(*)	(0.05)	(0.03)	(0.21)		(0.03)	(0.01)	(0.02)	(0.03)	(0.06)	(0.02)	(0.02)	(0.03)	(0.14)
	DOC∶DON	dry	25	41	21	7	dry	37	19	16	17	13	13	16	25	6
			(*)	(*)	(5)	(*)		(13)	(*)	(*)	(2)	(3)	(*)	(*)	(5)	(3)
	BDOC (%)	dry	39.6	16.9	7.7	26.1	dry	5.3	14.2	10.8	6.5	12.7	5.8	9.9	14.5	35.9
			(3.9)	(2.7)	(2.7)	(*)		(3.4)	(10.5)	(1.5)	(5.7)	(5.5)	(2.3)	(11.6)	(*)	(6.4)
	FI	dry	1.85	1.80	1.72	1.90	dry	1.60	1.87	1.76	1.77	1.69	1.72	1.74	1.62	1.95
			(*)	(*)	(*)	(*)		(*)	(*)	(*)	(*)	(*)	(*)	(*)	(*)	(*)
	SUVA (l/mg C cm)	dry	0.88	1.38	2.42	2.04	dry	2.28	0.69	0.50	0.75	1.11	1.29	0.36	1.40	0.30
			(*)	(*)	(*)	(*)		(*)	(*)	(*)	(*)	(*)	(*)	(*)	(*)	(*)
	I_C_/I_A_	dry	0.91	0.75	0.71	0.89	dry	0.55	0.63	0.42	0.54	0.38	0.38	0.49	0.58	0.63

Standard deviation is reported in brackets; (*) = standard deviation lower than 10%. (gw) = groundwater samples; (PIT) = Pond Isolation Time; (Q) = Discharge; (EC) = Electrical Conductivity; (CI) = Chemical Index; (BDOC) = Biodegradable Dissolved Organic Carbon; (FI) = Fluorescence Index; (SUVA) = Specific UV Absorbance; (I_C_/I_A_) = Ratio of intensities of C and A fluorescence peaks. See text for additional details.

On average, the descriptors used to characterize the dissolved organic matter (DOM) such as DOC∶DON ratio, percentage of biodegradable DOC (BDOC), and ratio of intensities of C and A fluorescence peaks (I_C_/I_A_, a measure of in situ microbial degradation), showed higher values in summer than in autumn (Table 1). In particular, DOC was more biodegradable in ponds (BDOC>16.9%) than in running waters (BDOC<14.5%). In summer, BDOC was directly related to DON (r = 0.92, df = 4, p<0.01,) and inversely related to CI (r = 0.84, df = 4, p<0.05). Moreover, I_C_/I_A_ showed higher values (>0.75) in ponds than in running waters and groundwater.

The Fluorescence Index (FI, a descriptor of DOM origin) value around 1.6, which is typically found in soil leachates in the Fuirosos basin [23], indicated a greater contribution of allochthonous DOC in the flowing waters than in isolated ponds. In summer, specific UV absorbance at 254 nm (SUVA, a measure of DOM aromaticity) values ranged from 0.88 to 2.42 with highest values in running water sites 4 and 7 and lowest values in groundwater and sites 2 and 3. In autumn, these values were lower than in summer and tended to increase with respect to CI values.

**Figure 3:**
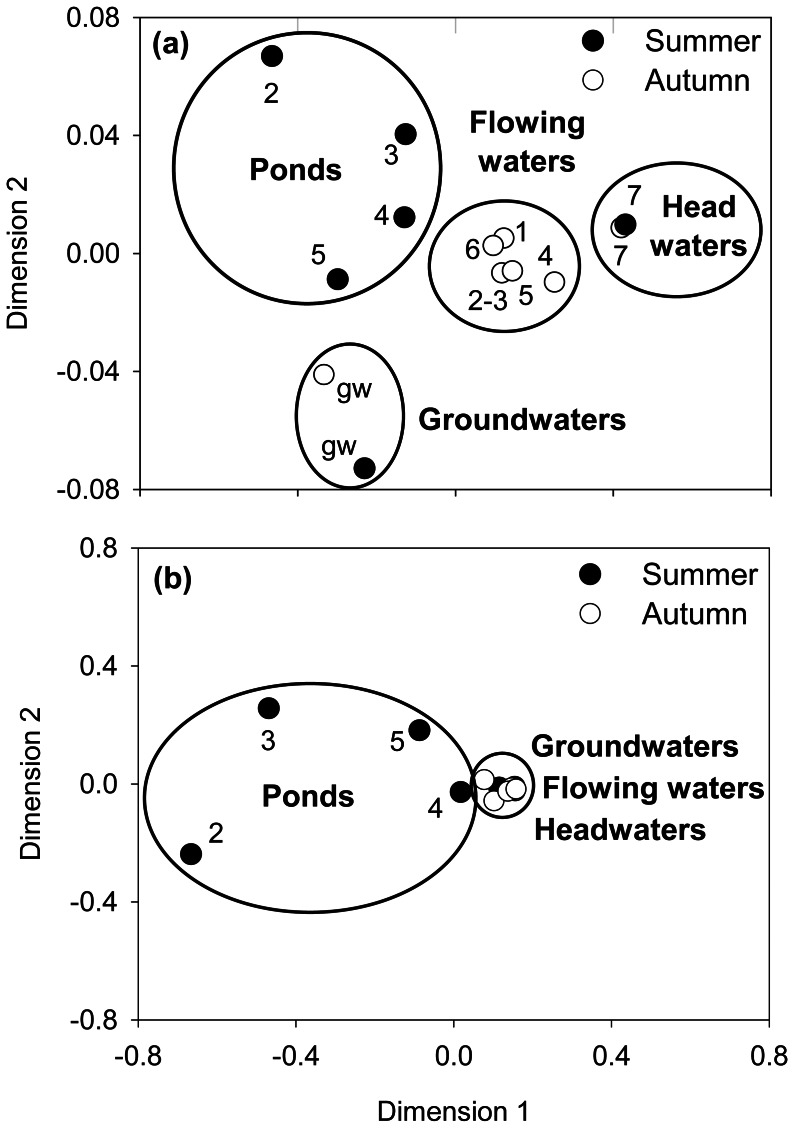
nMDS ordination analysis. (a) biogeochemical data as in Table 1, and (b) community composition analysed by CARD-FISH from both summer and autumn samplings.

Overall, hydrological fragmentation enhanced biogeochemical heterogeneity as graphically summarised by the non-Metric Multi-Dimensional Scaling analysis (nMDS). In the headwater and groundwater sites (7 and gw) changes between the two hydrological periods (summer and autumn) were minimal compared to the high biogeochemical shift in isolated ponds (Fig. 3 a).

### Bacterial community composition by in situ hybridization

**Figure 4:**
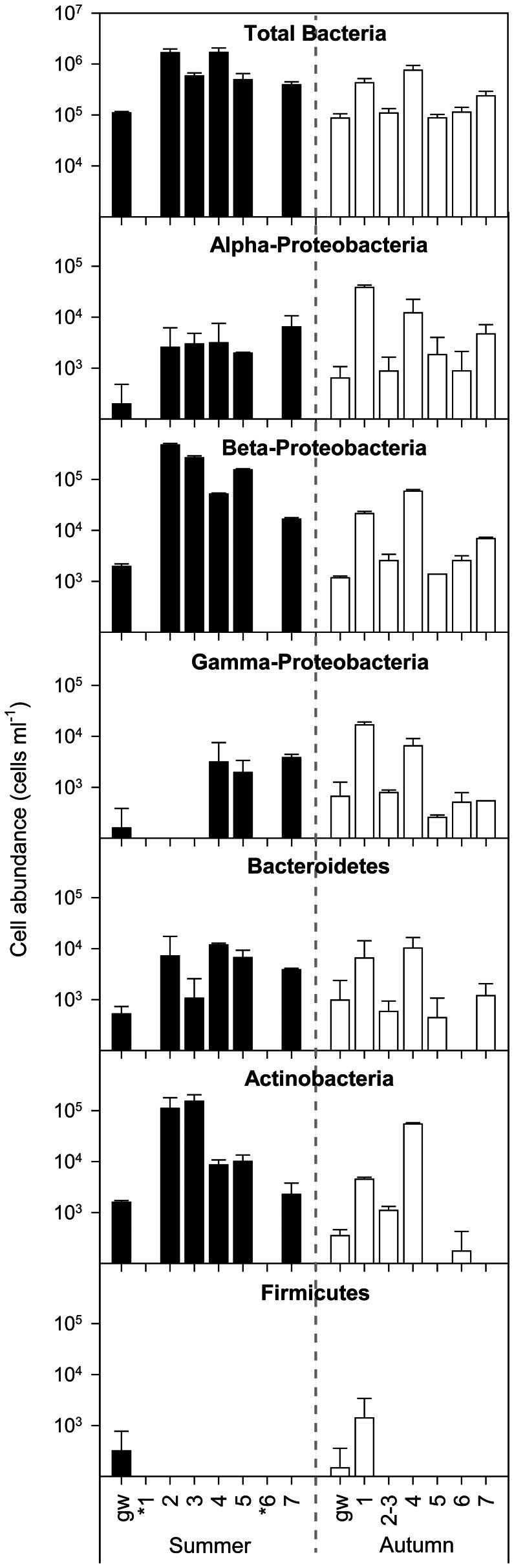
Bacterial abundance. Abundance of the selected bacterial phylogenetic groups as analysed by CARD-FISH. Values are expressed as cells per ml. Error bars indicate standard deviation. (*) = dry reach in summer.

Bacterial cell abundance averaged 9.7×10^5^ cells/ml during summer, with great variability among the sites (CV = 68%). In autumn, the cell abundance was lower (2.9×10^5^ cells/ml) and less variable (CV = 21%). Groundwaters (site gw) had the lowest concentrations at any time (average 9.8×10^4^ cells/ml, CV = 16%) (Fig. 4). We also observed statistically significant differences, both between seasons (two way ANOVA F = 569.03; P<0.001) and among the sites (F = 163.46; P<0.001). Pair-wise multiple comparisons (Student-Newman-Keuls method) revealed no significant differences between sites 2 and 4 or among sites 3, 5 and 7 in the summer. In autumn, cell abundance in site 4 showed the highest value, significantly different from any other site except site 1 (q = 1.709 p>0.05). Cell abundance was positively correlated with DOC concentration (r = 0.72; P<0.004) and water temperature (r = 0.51; P<0.05). The multiple regression analysis highlighted that the seasonal and spatial dynamics of cell abundance could be significantly explained by DOC, Temperature, and NH_4_ dynamics (r = 0.93; P<0.001).

When quantifying the occurrence of specific clusters, beta-*Proteobacteria* was the most abundant among the analysed groups in both the summer (up to 4.7×10^5^±0.4×10^5^ cells/ml in Site 2) and the autumn (up to 5.9×10^4^±0.4×10^4^ cells/ml in site 4). On average, *Actinobacteria* was the second-most abundant group, reaching the highest values in summer (isolated ponds 2 and 3). The alpha- and gamma-*Proteobacteria* showed the highest values in autumn at sites 1 and 4. The *Bacteroidetes* did not display a seasonal trend, and were below 1×10^4^ cells/ml, while the *Firmicutes* were only detected in groundwater samples and in site 1 during autumn (Fig. 4).

**Figure 5:**
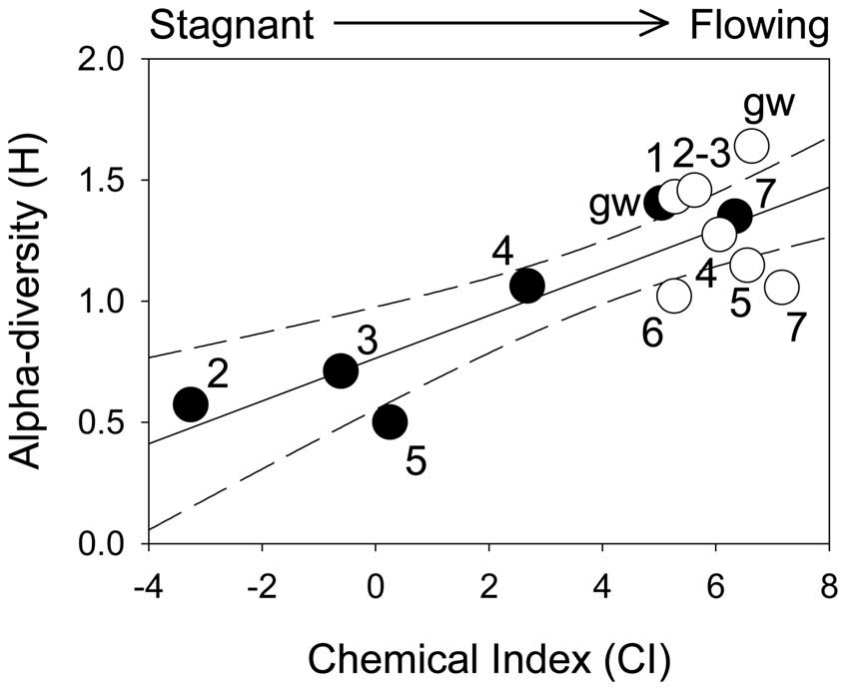
Chemical Index vs alpha-diversity. Relationship between biogeochemical conditions (expressed as Chemical Index – CI = log([O_2_]/[N-NH_4_])) and the alpha-diversity index (r = 0.82; p<0.05). s = summer; a = autumn.

The nMDS analysis revealed a consistent shift of community composition in the isolated ponds (Fig. 3 b). CI was the chemical variable most strongly associated with community composition (Mantel test r = 0.51; p<0.001) followed by qualitative DOM properties (i.e. intensity of fluorescence peaks A and C, r = 0.37 and r = 0.33 respectively; p<0.001). When considering two or more variables together, the explanatory power of the Mantel test did not improve. Overall, changes in the alpha-diversity level of the bacterial community were observed in summer (Shannon entropy, H = 1.35 in headwaters and H = 0.50 in isolated ponds), whereas in the autumn it increased from 1.06 to 1.46 along the stream continuum. We observed a significant linear relationship (r = 0.82; p<0.05) between CI and the alpha-diversity level of the bacterial community (Fig. 5).

### Bacterial phylogenetic assessment by fingerprinting and sequencing

**Figure 6:**
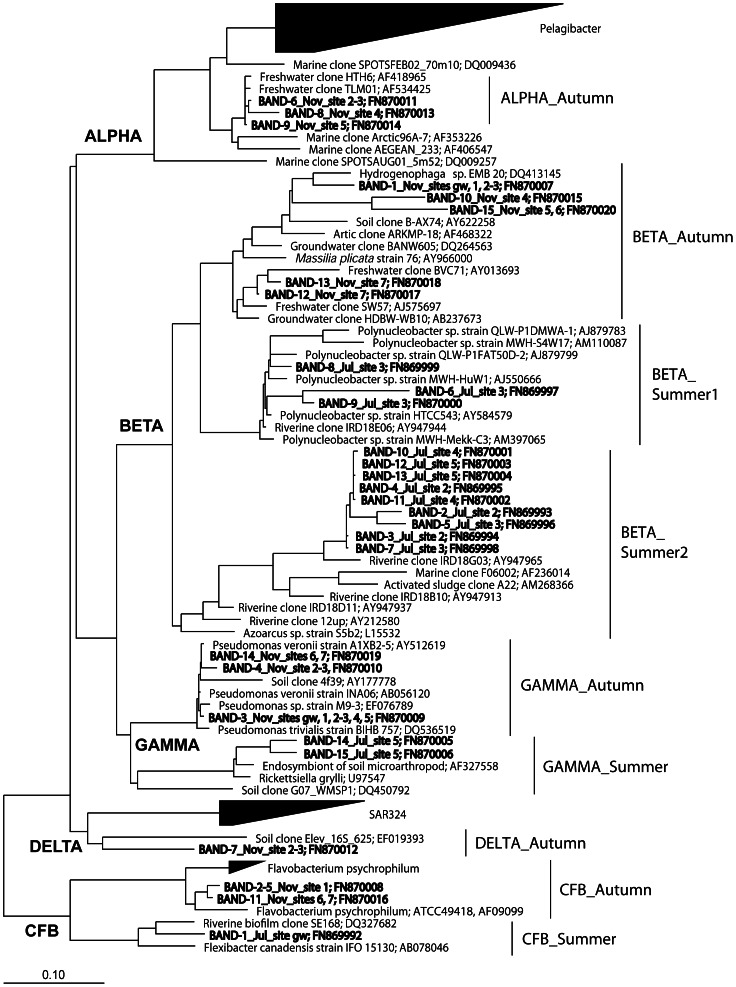
Maximum parsimony tree of the bacterial 16S rRNA gene sequences. Partial sequences were inserted into the optimised and validated tree available in the ARB program without changing the initial topology of the consensus tree provided by default. The scale bar represents 10% estimated divergence. Those identical sequences found in different sites are indicated in the band code. ALPHA, BETA, GAMMA, DELTA = Classes of *Proteobacteria*; CFB = *Bacteroidetes*.

The number of DGGE bands was similar among samples and ranged between 8 and 13. The prominent bands (overall c.a. >80% of total band intensity in the lanes) were sequenced and alpha-, beta-, gamma-, and delta-*Proteobacteria* and *Bacteroidetes* were identified (Fig. 6). DGGE detects populations with a relative abundance around 0.1–1% of the total PCR-targeted cells [24], and we cannot, therefore, disregard the possible presence of other populations below this detection limit. It was possible to detect a shift in the retrieved phylotypes between summer and autumn samples for each bacterial phylum by grouping the samples according to the sampling season (Fig. 6).

The alpha-*Proteobacteria* (freshwater SAR11 cluster) were only detected in autumn as very prominent DGGE bands in sampling sites 2, 3, 4 and 5. The beta-*Proteobacteria* were abundant in most of the sites and split into three groups for the two different sampling periods: i) beta_Autumn, detected only in the autumn samples and related to *Hydrogenophaga* and *Massilia* spp.; ii) beta_ Summer1, detected in the summer samples and closely related to *Polynucleobacter* sp.; and iii) beta -Summer2, detected in the summer samples and distantly related to *Azoarcus* sp. (see accession numbers in Fig. 6). Overall, beta-*Proteobacteria* were heterogeneously distributed both in space and in time for the different sampling points.

We also observed changes in population composition between the two sampling periods for gamma-*Proteobacteria* (closely related to *Pseudomonas* sp. in autumn and to *Rickettsiella* sp. in summer) and *Bacteroidetes* (closely related to *Flavobacterium* spp. in autumn and to *Flexibacter* spp. in summer). The gamma-*Proteobacteria* were widespread in autumn, but were only detected in site 5 in summer. The delta-*Proteobacteria* (distantly related to the SAR324 cluster) were only detected in autumn. *Bacteroidetes* were found in a few sites in autumn (sites 1, 6, and 7) and only in site gw in the summer.

## Discussion

In the Mediterranean region, hydrological fragmentation is a common feature of both headwaters and extended reaches of large rivers [25]–[26]. When stream flow is interrupted, the longitudinal lentic water continuum disappears. The water mass remains fragmented and stagnant in ponds and the hydrological connections to the surrounding environment are lost [27]. Our results showed that the qualitative DOM characteristics were extremely sensitive to the hydrological conditions showing a higher contribution of autochthonous organic solutes in the disconnected ponds in summer. Furthermore, the biogeochemical status of each disconnected pond was clearly related to the length of the isolation period. Hence, the generation of a set of transient isolated patches with high rates of biogeochemical processes [28], [29] and with different isolation times, increases the chemical heterogeneity spectrum of the entire Fuirosos stream system during summer [30].

The effect of biogeochemistry on microbial community composition and functioning is well known [31]. In the present study, the aquatic bacterial communities responded promptly to the biogeochemical heterogeneity. We observed that total bacterial abundance was positively related to DOC and N-NH_4_ concentrations and to temperature, with the highest values in the isolated ponds, where DOM mainly originated from in-situ microbial processes (i.e. high FI and Ic/Ia values). The alpha-, beta- and gamma-*Proteobacteria* accounted for the largest number of bacteria among the analysed groups, in agreement with the findings of other studies carried out in riverine systems by either 16S rDNA gene sequencing or FISH counts [32]–[34]. The dominance of beta-*Proteobacteria* in freshwater habitats was highlighted in a variety of other investigations [3], [22]. The alpha and gamma classes were consistently less abundant than the beta-*Proteobacteria*, and were also associated with more oxic conditions. Members of *Bacteroidetes*, reported as an autochthonous component of the limnetic habitat [32], were found in lower numbers. As shown by earlier investigations, the proportion of *Actinobacteria* can vary considerably in different freshwater habitats [35] and may locally belong to the dominant fractions of freshwater bacterial communities. *Firmicutes* were only locally present in low-stream reaches and groundwater with a low relative abundance, as reported for other stream environments [32], [36], [37]. The distribution of these bacterial classes showed relevant changes between stagnant and flowing waters. The degree of aerobic/anaerobic conditions seemed to be the main driver of the bacterial community composition and it was related to PIT and to autochthonous DOM. In summer, the bacterial communities showed higher alpha diversity in the stream reaches with flowing water. In the pond most recently isolated (site 5, PIT = 7 days), the community was composed by all the groups primarily found in headwaters, although a reduction in the abundance of alpha- and gamma-*Proteobacteria* was already evident. As isolation time increased (PIT>10 days) and CI decreased (Sites 2, and 3), the abundance of the beta-*Proteobacteria* and *Actinobacteria* increased significantly. These groups were probably adapted to the selective conditions that follow the interruption of the flow. Overall, during the summer, the change in community composition was closely related to the chemical variability determined by the isolation time. In autumn, as flow was restored, the alpha diversity of the communities increased downstream. This could be related to the greater contribution of organic substances derived from the allochthonous DOM in the upper reaches and the autochthonous DOM in the downstream reaches, as indicated by the increasing FI values. Therefore, when the river continuum was re-established, organic C transport provides a linkage along the stream that is fundamental to the nature of fluvial systems [38].

Variations in bacterial species composition, characterised by PCR-based community fingerprinting techniques, are often related to physical, chemical, and biological factors (e.g. [11], [39], [40]). Microcosm experiments clearly demonstrated that variations in DOM composition and origin (allochthonous versus autochthonous) could affect community composition [31], [41], [42]. In our study, the analysis of the genetic fingerprinting showed a temporal clustering instead of a spatial grouping. This indicated a seasonal shift of the populations within the same phylogenetic group (e.g. beta-, gamma-*Proteobacteria* and *Bacteroidetes*) in the transition from summer to autumn conditions for most of the sites. The sequences of beta-*Proteobacteria* retrieved from the hypoxic summer ponds were mainly affiliated to two different clusters, which were closely related to the genera *Azoarcus* spp. and *Polynucleobacter* spp., while the groups found in autumn were related to *Hydrogenophaga* and *Massilia* spp. The genus *Azoarcus* is reported to degrade aromatic compounds under denitrifying conditions [43], [44], and it might contribute to the removal of nitrogen and aromatic DOM in the isolated ponds. It is likely that *Polynucleobacter* spp. is ubiquitously distributed in lentic and stagnant habitats worldwide [45]. Recent observations from laboratory studies suggest that they prefer autochthonous rather than allochthonous substrate sources [46], [47]. Our results, therefore, confirm that the source and lability of DOM could drive bacterial community composition [48].

In conclusion, we found that (i) the temporary hydrological fragmentation simultaneously reduced the microbial dispersion and affected local environmental characteristics (i.e. redox regime and DOM quality) prompting the gradual development of selected bacterial groups in isolated water ponds, and (ii) a shift of the populations within the same phylogenetic classes (i.e. beta- and gamma-*Proteobacteria*) was observed in the transition from summer to autumn conditions. In the context of the seasonal dynamics, the temporary limitation in microbial dispersal and the environmental changes, promoted by pond isolation, gradually revealed local patterns in the community composition.

## Materials and Methods

All the sampling sites along the Fuirosos stream are located in the protected area of Natural Park Montnegre-Corredor, under the authority of the Diputació de Barcelona. No specific permissions were required for all these locations to carry out the research activities, reported in this study, by the University of Barcelona. We confirm that the field studies did not involve endangered or protected species.

### Site description and sampling

Fuirosos, a tributary of the River Tordera, is a third-order stream that drains a forested granitic catchment area of 16.2 km^2^ (NE Spain, 41°42′N, 2°34′W, 50–770 m a.s.l.). The climate is Mediterranean, with mean monthly temperatures ranging from 3°C in January to 24°C in August. Precipitation mostly falls in the autumn and spring, with occasional summer storms. The average annual mean rainfall for the region is 750 mm [49], and the catchment is covered mainly by perennial woodland, with agricultural fields representing <10% of the area.

The mean daily flow at the hydrochemical long-term monitoring station (Site 1, Fig. 1) ranged between 0 and 20 L s^−1^ [50]. During summer, the basal discharge decreased from 15 L s^−1^ in May to 0 L s^−1^on June 6th, when the water flow stopped and the water masses started to be confined in ponds. During this period, daily hydrological monitoring along the stream network allowed us to estimate the age of each isolated water pond. Surface flow recovered in the fluvial network on August 24th and the basal discharge gradually increased to 3–4 L s^−1^ and remained steady at around these values throughout November. In order to cover a wide range of hydrological conditions, samples were collected during summer (July), when the stream was disconnected into a series of isolated ponds, and after autumnal rainfall (November) when the stream connectivity was re-established. During the summer sampling, the entire stream network was completely dry, except in the five ponds where water was collected (ponds 2, 3, 4, 5, 7; Fig. 1 and 2). Ponds 2, 3, 5 were completely isolated, with stagnant water since 15, 12 and 7 days before sampling respectively. Ponds 2 and 3 were 15 meters apart. In ponds 4 and 7 water was still flowing (0.1–0.2 L s^−1^). During the autumn sampling, when the surface flow was continuous along the fluvial network (discharge 3–4 L s^−1^), samples were collected in six locations including those previously sampled in summer (Fig. 1 and 2). In autumn, only one sample was collected in correspondence of the summer ponds 2 and 3 as no differences were registered in water physical and chemical in situ measurements in the two contiguous sites.

Samples were also collected from a shallow, perched, riparian aquifer (site gw), which was recharged by stream water, by a peristaltic pump from a well (2 m depth) located outside the stream edge that perforated the sandy-gravel and the weathered granite layers [51]. We decided to sample this aquifer because during dry periods, it loses the hydrological connectivity with surface water, essentially becoming an underground pond with stagnant infiltrated water [27]. It was not possible to determine the PIT value for the groundwater site. However, it can be presumed that it was isolated from the surface's running water for at least 15 days before the sampling date.

Overall, the sampling sites covered a hydrological gradient including: surface stagnant waters in isolated ponds (sampling points 2, 3 and 5 in summer); surface waters under very low-flow (sampling points 4 and 7 in summer) and basal-flow conditions (sampling points 1 to 7 in autumn); and groundwater (gw) (Fig. 1 and 2). Temperature, pH, electrical conductivity, oxygen concentration and, when possible, discharge were measured at each sampling location. Triplicate water samples were collected in clean acid-washed bottles for water chemical characterisation (2 L) and in sterile flasks for microbiological analysis (1 L). The water samples were pre-filtered in the field with pre-combusted GF/F filters (Whatman) and transported in an icebox (4°C).

### Water chemistry

Chloride and sulphate content was analysed with liquid chromatography using a Metrohm 76 compact IC, while nitrates and ammonia were determined colorimetrically using a Technicon auto-analyser, by means of the Griess-Ilosvay method [52] and oxidation by salicylate [53], respectively. The relationship between the concentration of dissolved oxygen and nitrogen, in ammonium form (CI = log([O_2_]/[N-NH_4_]) was used to describe the degree of the aerobic/anaerobic conditions in the waters bodies [30]. Under anaerobic conditions, CI is expected to have low values as a consequence of the decrease of oxygen and the concomitant accumulation of reduced solutes (i.e. NH_4_) [54], [55].

DOC and total dissolved nitrogen (TDN) concentrations were determined using a Shimadzu TOC-VCS with a coupled TDN analyser unit. Meanwhile, DON was estimated from the difference between the TDN and the dissolved inorganic nitrogen. Five descriptors were used to characterise the DOM: DOC∶DON ratio, BDOC, SUVA, FI, I_C_/I_A_. The BDOC content was determined according to the method proposed by Servais et al. [56], using site 4 GF/F filtered water as inoculum in all samples. SUVA was calculated from the measured absorbance at 254 nm, corrected by the cuvette path length in meters and the DOC concentration. Previous studies have revealed that SUVA is highly correlated to DOM aromaticity [57], [58]. Fluorescence measurements were performed using a Shimadzu RF-5301PC. The FI, calculated from the ratio between the emission intensities at 450 and 500 nm at a fixed excitation of 370 nm, allows discriminating DOM origin [59]. It ranges from 1.2 to 2, where low values indicate allochthonous origin while high values point to an autochthonous origin. The EEMs were obtained by concatenating emission spectra ranging from 280 to 690 at a range of excitation wavelengths from 240 to 420 in steps of 10 nm. Raman scattering was corrected by subtracting the value of ultra pure water blanks, and the EEMs were then corrected by the Raman area. Fluorescence intensity is expressed in Raman units. The determination of fluorescent peaks was performed by visual “peak picking”, using the coordinates estimated by Coble [60] as reference. The fluorescence maximum of peaks C and A were used to calculate I_C_/I_A_ [30]. Fluorescent peaks A and C are generally associated with substances of terrestrial origin [60]. However, the fluorescence of certain components, which correspond to peak C, increases as a result of the microbial degradation of estuarine DOM of an autochthonous origin [61]. Accordingly, high values in this ratio provide information about the magnitude of the in-situ microbial processing.

### Fluorescence in situ hybridization

CARD-FISH was performed following the protocol optimised by Fazi et al. [62], [63]. The following rRNA-target HRP-labelled probes (Biomers, Ulm, Germany) were used: ALF968, targeting sequence types affiliated with alpha-*Proteobacteria*; BET42a for beta-*Proteobacteria*; GAM42a for gamma-*Proteobacteria*; CF319a for *Bacteroidetes* (formerly *Cytophaga-Flavobacterium-Bacteroides*); HGC69a for *Actinobacteria*; LGC354a for *Firmicutes* [64]. The stained filter sections were inspected on a Leica DM LB 30 epifluorescence microscope (Leica Microsystems GmbH, Wetzlar, Germany) at 1000× magnification. At least 300 cells were counted in >10 microscopic fields randomly selected across the filter sections. The relative abundance of hybridized cells was estimated as the ratio of hybridized cells to total DAPI-stained cells.

### DGGE fingerprinting analysis and 16S rRNA gene sequencing

A variable volume (200 ml to 800 ml) of stream water was filtered through a number of 0.2 µm- polycarbonate membranes (Nuclepore) until filter clogging decreased the flow rate to <1 ml per min. Because of the differences in cell abundance among sites, filter clogging provided an indication that we had processed a similar number of cells for each sample. Samples were digested in lysis buffer and phenol extracted [65], [66]. The 16S ribosomal RNA gene was PCR amplified with the universal bacterial primer set 341fGC-907r and run in a DGGE as previously described [67]. Prominent bands were excised from the gel, re-amplified, and sequenced [68]. The sequences were submitted to a BLAST search [69] and inserted into an optimised and validated ARB consensus tree (www.arb-home.de). The 16S rRNA gene sequence accession numbers at EMBL are from FN869992 to FN870020.

### Statistical analysis

The abundances of different phylogenetic taxa estimated by CARD-FISH in each sampling site were compared by performing a two-way ANOVA and pair-wise multiple comparisons (Student-Newman-Keuls method). A multiple regression analysis was run to identify the environmental parameters that best explained the variability of the abundance of the bacterial taxa. To explore similarities between the sampling sites along the gradient from flowing to stagnant waters, the nMDS was performed with log transformed data according to the basic Euclidean distance matrix. The analysis was computed with either the environmental variables or the abundances of the bacterial taxa estimated by CARD-FISH. In addition, Mantel tests were run (permutation tests for correlation between Euclidean similarity matrices; 1000 randomised runs) to determine which combinations of environmental variables were more closely related with the similarity patterns of the abundances of the bacterial taxa [70]. Multivariate analyses were performed by the PAST software package (PAlaeontological STatistics, ver. 2.05). The alpha-diversity index was based on the relative abundance of the phylogenetic taxa estimated by CARD-FISH [Shannon entropy, H = −Σ (Pi ln Pi), Pi = relative abundance].

In order to evaluate if seasonal (summer versus autumn) community changes predominate over episodic habitat fragmentation, the primary DGGE bands at the same position in the different lanes of the gel were identified. A binary matrix (1/0) was produced to build a dissimilarity matrix based on the Jaccard coefficient (Sj) and a dendrogram with the un-weighted pair group average linkage method (UPGMA). Relative band intensities were calculated by comparison with the total intensity of all bands in each lane [68].
